# Supplementary material for: Causal interplay between lactose intolerance and gut microbiota: a combined bidirectional Mendelian randomization and in vivo validation study
Source: Front Nutr. 2026 Jun 1;13:1803337. doi: 10.3389/fnut.2026.1803337 (PMC13265576; doi:10.3389/fnut.2026.1803337)
Supplement: Supplementary file 1 [file Data_Sheet_1.zip › supplementary materials/Forward/leave one out cross validation/ebi-a-GCST90027751.finngen_R12_E4_LACTONAS.pdf]

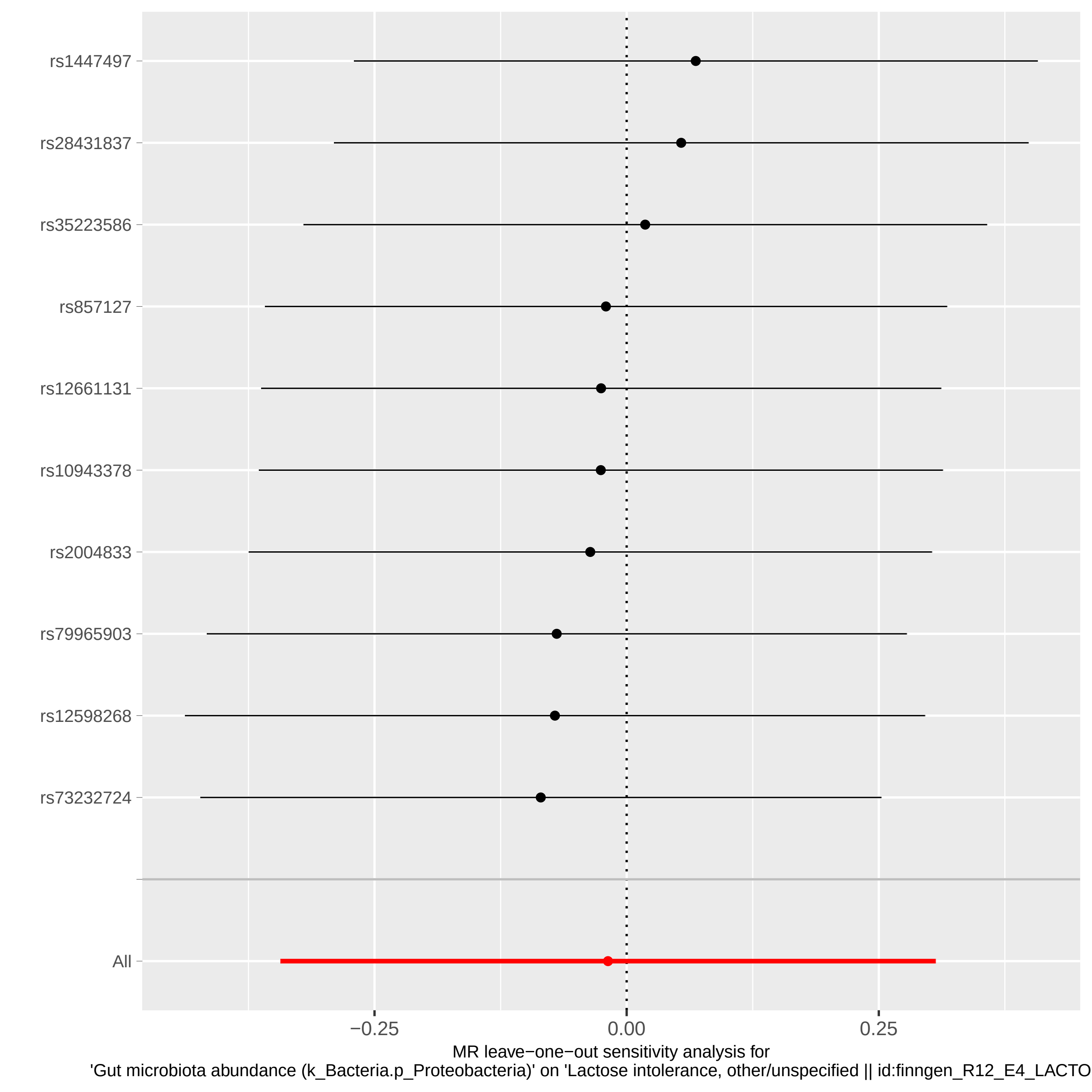

rs1447497
rs28431837
rs35223586
rs857127
rs12661131
rs10943378
rs2004833
rs79965903
rs12598268
rs73232724
All
−0.25
0.00
0.25
MR leave−one−out sensitivity analysis for
'Gut microbiota abundance (k_Bacteria.p_Proteobacteria)' on 'Lactose intolerance, other/unspecified || id:finngen_R12_E4_LACTO
